# Not all lymphoid aggregates in chronic lymphocytic leukemia (CLL) patients are due to CLL!

**DOI:** 10.1002/ccr3.3701

**Published:** 2021-01-08

**Authors:** Lily Mahapatra, Tianjiao Wang, Yi‐Shan Lee, John L. Frater

**Affiliations:** ^1^ Department of Pathology and Immunology Washington University School of Medicine St. Louis MO USA

**Keywords:** *Anaplasma*, chronic lymphocytic leukemia, *Ehrlichia*, ehrlichiosis, tick borne illness

## Abstract

Infection is a common cause of morbidity and mortality in chronic lymphocytic leukemia and should be considered when examining bone marrow specimens to identify a potentially treatable pathogen.

## Graphical abstract

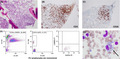

A 75‐year‐old man with a history of chronic lymphocytic leukemia (CLL) diagnosed in 2015 and currently treated with ibrutinib presented to the emergency department with a 1‐month history of malaise and generalized fatigue. The patient denied any recent travel history and had no known sick contacts. A computed tomography scan showed hepatosplenomegaly and extensive lymphadenopathy. A complete blood count showed bicytopenia; white blood cell count, 7.8 × 10^9^/L; hemoglobin, 12.3 g/dL; platelets, 39 × 10^9^/L. Additional laboratory findings included elevated liver enzymes: AST, 81 U/L; ALT, 72 U/L; and elevated alkaline phosphatase, 353 U/L.

Given the patient's past medical history of CLL, a bone marrow biopsy was performed and showed hypercellular marrow with multiple lymphohistiocytic aggregates (Figure 1A) that mimicked CLL. However, the aggregates were predominately composed of T cells rather than B cells, highlighted by CD3 and CD20 staining (Figure 1B,C), favoring a reactive process. Flow cytometry demonstrated low‐level involvement by CLL, with approximately 3% monoclonal, kappa‐restricted B lymphocytes (Figure 1D). Review of the bone marrow aspirate showed myeloid precursors with intracellular morulae (Figure 1E) and ehrlichiosis was confirmed by detection of *Ehrlichia* species DNA by polymerase chain reaction. Morulae were not identified in the patient's peripheral blood smear; however, it has been documented that morulae can be seen in only 22% to 38% of peripheral blood smears from ehrlichiosis patients.^1^, ^2^ Upon further discussion, the patient disclosed that he recently hiked in the woods of rural Missouri and received numerous tick bites. The patient was treated with doxycycline 100 mg two times per day for 10 days and clinically improved.

**FIGURE 1:**
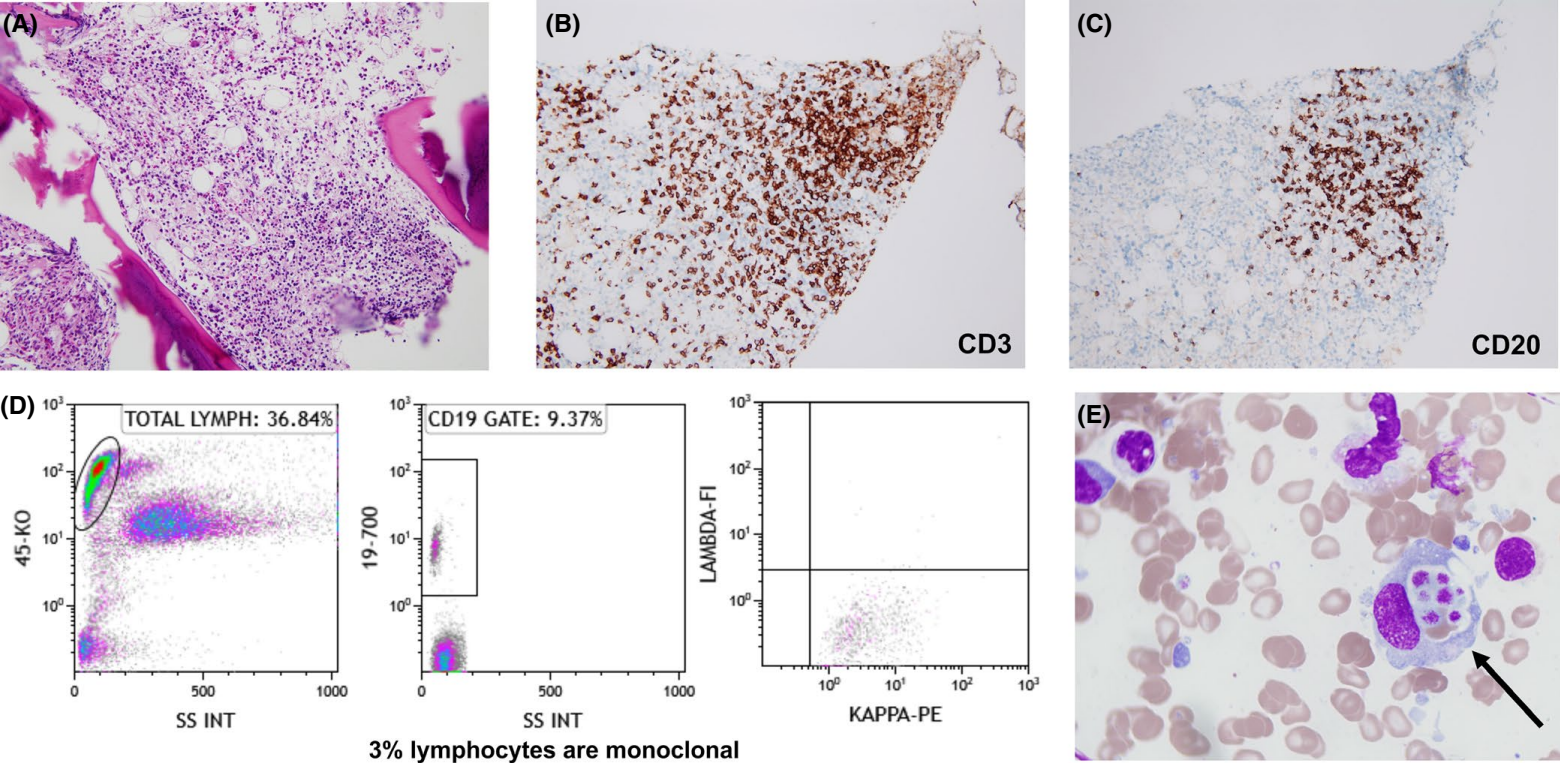
A, Bone marrow trephine biopsy showing lymphohistiocytic aggregate (hematoxylin and eosin, original magnification ×400). Immunohistochemistry demonstrating admixed CD3 (B) and CD20 (C) positive lymphocytes in the aggregate. D, Flow cytometric analysis of the bone marrow aspirate revealed a monoclonal B‐cell population comprising ~3% of specimen cellularity (E) Review of the bone marrow aspirate revealed morulae of *Ehrlichia* organisms in granulocyte precursors

There have been very few reports of bone marrow findings in patients with ehrlichiosis, but lymphohistiocytic aggregates have been described.^2^ This unique case highlights the importance of laboratory testing supported by a thorough clinical history to ensure accurate diagnosis.

## CONFLICT OF INTEREST

None declared.

## AUTHOR CONTRIBUTIONS

LM: wrote the manuscript. TW: acquired data. Y‐SL: analyzed and interpreted data. JLF: analyzed and interpreted data and wrote the manuscript.

## ETHICAL APPROVAL

The manuscript was published with written consent of the patient. The manuscript complies with the ethical approval requirements.

## Data Availability

The data that support this study are available from the corresponding author upon reasonable request.
